# Interactions between worker ants may influence the growth of ant cemeteries

**DOI:** 10.1038/s41598-020-59202-0

**Published:** 2020-02-11

**Authors:** Tomoko Sakiyama

**Affiliations:** Department of Information Systems Science, Faculty of Science and Engineering, Soka University, Tokyo, 192-8577 Japan

**Keywords:** Computational biology and bioinformatics, Mathematics and computing

## Abstract

When an ant dies within a nest, a worker ant carries its corpse away from the nest and drops it onto a pile known as an ant cemetery. These ant cemeteries form cluster patterns, and the dynamics of the corpse piles have been studied experimentally. The aim of the present study was to investigate how sensitivity to the presence of nest-mates would influence the corpse-carrying behaviour of ants, and how this would impact the dynamics of corpse pile clustering. This was achieved by developing an agent-based computational model in which simulated ‘ants’ (the agents) carry and drop ‘corpses’, resulting in the growth of the corpse pile. In the model, the probability of an ant dropping a corpse was tuned according to the presence or absence of nest-mates. The pile dynamics of the resulting model showed a partial match with the time series evolution of corpse piles observed with real ants in previous experimental studies. Although the switch of probabilities is a thought experiment, our results suggest that the corpse-carrying behaviour of worker ants might be influenced by interactions with their nest-mates because there is evidence that ant behaviour can be influenced by encounter rates.

## Introduction

The collective behaviour of groups of ants seems to be the result of simple mechanisms at the agent level. This is an example of emergent behaviour, wherein the growth or evolution of more complex forms arises from simple rules. Studies have shown that the dynamics of ant systems at the macro-level can evolve via self-organising construction by worker ants (ants that perform tasks such as tending to the nest and looking after the larvae)^1–6^. Ants form various clustering patterns, which have been studied as examples of collective behaviours^4–8^, and they are known to aggregate many types of objects, including seeds, larvae and the corpses of their dead workers^9–11^. These clusters can form in the presence of environmental heterogeneities.

Necrophoresis is a behaviour observed in social insects in which the corpses of dead members of the colony are carried away from the nest; this prevents infection spreading through the colony^12^. Necrophoresis by worker ants can cause the formation of piles of dead ants known as ant cemeteries. Through a combination of experiments and modelling, this phenomenon has been rigorously demonstrated to be the result of simple clustering rules. The worker ants pick up corpses, carry them away from the nest and then drop them to form piles, resulting in the formation of several clusters. Interestingly, surviving clusters have been observed to undergo sigmoidal growth, with some clusters growing while others disappearing, which suggests that cluster formation can be autocatalytic^13^. Observation has revealed that worker ants drop or pick up corpses according to the local density of corpses. An ant carrying a corpse drops that corpse on a specific cluster with a probability that increases with the cluster size; thus, clusters grow through self-enhancement^13^. This phenomenon is an example of stigmergy, a mechanism that mediates agent–environment interactions. Stigmergy has been extensively studied in social insects^14–17^. Stigmergy can explain indirect task coordination in which the actions of individual agents are stimulated by a trace left in the environment; examples include the construction of termite mounds and digging activity by leaf-cutting ants. This way, individual actions indirectly influence the future repetition of those actions^15–17^. With the ant cemeteries, the ants construct the clusters via a form of indirect communication, with the cluster size influencing the probability an ant will drop the corpse it is carrying. Stigmergy can be a form of self-organisation, and it can produce collective, complex and intelligent structures.

Studies have suggested that the behaviour of ants can also be determined by earlier direct interactions with their nest-mates^18–21^. For example, harvest ants perform midden work by carrying objects such as a dead ant or an item of refuse to a pile, where they sort them. They seem to engage in this behaviour more often following encounters with other midden workers, with which they interact via brief antennal contacts^20^. The activities of worker ants are facilitated and regulated by interactions with nest-mates, and this can also contribute to the collective behaviours of colonies^18,19^. Pattern formation by worker ants can be coordinated by ant-to-ant interactions, with an individual ant’s contribution increased or reduced through interactions with nest-mates^21^. High encounter rates increase the probability of an ant performing a particular work activity. In this way, the collective building behaviours of insects can be influenced by their nest-mates; examples include the collective nest excavation of leaf-cutting ants and tunnel excavation by termites^22,23^.

Simulations, such as agent-based computational models, have been widely used to investigate the collective behaviours and decision-making processes of insects, including ants^24–28^. This methodology has been applied to the formation of ant cemeteries, with the development of an activator–substrate model, a complex function describing the time evolution of a local cluster of corpses^13^. Models such as this assume that ants can detect the number of corpses within their detection areas; this seems to have a direct influence on the explosive increase in pile size. The dropping rate per corpse-carrying ant seems to increase with the number of corpses detectable by the ant, reaching an asymptotic value. However, it is unlikely that an ant is able to estimate that value accurately. Simpler mechanisms might make more sense; for example, an ant might drop a corpse it is carrying based on a threshold number of corpses, following a step function. Threshold models are often proposed to describe the emergent behaviour of social insects^29,30^.

The aim of this study was to develop an agent-based model that describes the growth of ant corpse piles. It hypothesises two criteria for determining whether an ant drops the corpse it is carrying: the ant encounters more than a threshold number of corpses, and there is at least one nest–mate present locally. Thus, even if an ant detects the threshold number of corpses, it still tends to leave that location without dropping the corpse if its nest-mates are locally absent. This second criterion fills a gap between the dropping rate obtained directly from a threshold function and the experimentally observed dropping rate per corpse-carrying ant. For the sake of simplicity, these criteria were tested by developing a one-dimensional pile growth model in which the simulated ants could only move in two directions and corpse piles and more than one ant could occupy the same position. The results of this model were in agreement with aspects of those found in experimental studies of ant cemeteries.

## Methods

### Space, agents and model elements

The model simulated agents that move in a one-dimensional discrete lattice. This is the simplest arrangement but is not an unrealistic condition^13^. Agents (n = *Φ*) and corpses (n = *φ*) were randomly distributed within the field, and the positions of the agents were all updated synchronously. As boundary conditions, periodic boundaries were assumed.

The agents moved with a random walk-like motion (at a constant speed, without resting) and corpse pick-up and dropping behaviour was influenced by the number of corpses at the present location. To simplify the calculations, corpse clusters and piles were considered to be tower-like objects at a single discrete location. A cluster of corpses was defined as a pile if it consisted of five or more corpses. Piles could grow only through the aggregation of clusters. In the initial conditions, scattered clusters were distinguished from piles.

### Model development

Two models were developed: the Interaction Model, in which agent behaviour was influenced both by the number of corpses at a location and by nest–mate interactions, and the Threshold-only Model, in which agent behaviour was determined only by the number of corpses at a location. The latter model served as the control model. The models were coded in the C programming language. Pseudocode for the two models is available in the supplementary file.

**Figure 1:**
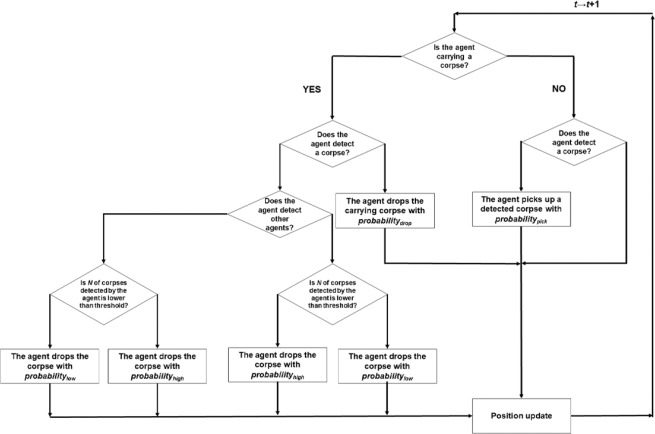
Flowchart for the Interaction Model. The left side of each bifurcation represents “YES” and the right side represents “NO”.

The Interaction Model was implemented as follows. As the initial condition, all the agents are non-carrying (i.e., not carrying corpses). At each time step, if a non-carrying agent detects a corpse in its current location, it picks up the corpse with probability *probability*_pick_. Conversely, a carrying agent will drop the corpse at a corpse-empty location with fixed probability *probability*_drop_; this probability has a small value to indicate that agents rarely drop a corpse away from a cluster. If one or more corpses are already present at a location, the carrying agent drops the corpse according to one of two probabilities according to the number of corpses and the presence of other agents at the location (Fig. 1). If another agent is present at the location, the carrying agent drops the corpse with *probability*_high_ if the total number of corpses at the location is higher than the threshold or *probability*_low_ if the number is lower than the threshold. Thus, a carrying agent is more likely to drop the corpse where the corpse threshold has already been exceeded. If there is no other agent at the location, these probabilities are switched: the corpse is dropped with *probability*_low_ if the threshold is exceeded but with *probability*_high_ if there are fewer corpses than the threshold at the location. Thus, in the absence of other agents, the corpse is less likely to be dropped at a location where the number of corpses already exceeds the threshold.

The switch of probabilities is, in effect, a thought experiment. It has no biological underpinnings; there is no direct evidence that ants modify the probability of dropping corpses according to the presence of nest-mates. However, as noted earlier, there is evidence that behaviours can be influenced by encounter rates^18^. In the Interaction Model, encounters with other worker ants are represented as other agents being present at the current position. When that is the case, an agent carrying a corpse will tend to drop the corpse more often when the cluster of corpses at their location is above a threshold size. Conversely, if an agent carrying a corpse does not detect any other agents nearby, in this model it is less likely to drop the corpse even when the local cluster of corpses exceeds the threshold; however, it is more likely to drop the corpse when the cluster is small to stop working, demonstrating that worker agents tend not to engage in the current work when no other agents are present in the current position. Although there is no biological evidence for these assumptions, they describe plausible ant behaviours, and the switched probabilities in the model seem to serve as a key effect in the evolution of the growth of clusters and piles.

The Threshold-only Model is identical to the Interaction Model except that the carrying agent at a location where one or more corpses are present always drops its corpse with *probability*_high_ if the total number of corpses at the location is higher than threshold and *probability*_low_ if the number is lower than the threshold, regardless of the presence of other agents at the current location. Thus, the model is based only on a simple threshold.

### Parameter values

The parameters used in the models are listed in Table 1. Values for the ratio of the number of corpses to the field length (*φ*/*l*), *Probability*_high_ and *Probability*_low_ were based to some extent on the results of a previous study^13^. This suggested that the probability of dropping for corpse-carrying ants was in the approximate range 0.10–0.50, but with low rates (<0.10) when the size of the pile was close to zero. For this reason, *Probability*_drop_, which represents the probability of a drop at a corpse-empty location, was set to 0.05. The probability of picking up a corpse appeared to decrease as the size of the pile increased, with a value of ≈0.04 at most. For simplicity, the parameter *probability*_pick_ was set to 0.05. The sensitivity of the model to changes in the parameter values for *φ* and *l* was also tested.

**Table 1:** Parameters used in the models.

Parameter	Value	Description
*Φ*	100	Number of agents
*φ*	400	Number of corpses
*l*	100	Field length
*Probability*_high_/*Probability*_low_	0.5/0.1	High and low probabilities of dropping a corpse at a location where one or more corpses are already present
*Probability*_pick_	0.05	Probability of picking up a corpse
*Probability*_drop_	0.05	Probability of dropping a corpse at a corpse-empty location
*threshold*	10	Threshold value for choosing *Probability*_high_ or *Probability*_low_
*N of time step*	10,000	Number of time steps for the measurement
*N of trials*	100	Number of trials

## Results

**Figure 2:**
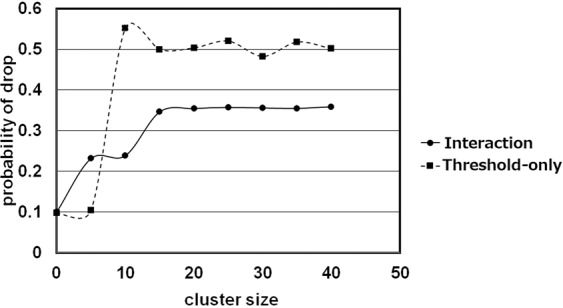
The relationship between the probability of dropping a corpse and cluster size in each model. The data are mean values for 100 trials.

Differences between the results of the Interaction and Threshold-only Models were investigated. The Interaction Model resulted in intermediate-size clusters of corpses (i.e., those with fewer than 20 corpses) that were significantly larger than those with the Threshold-only Model (4.13 vs. 1.93 corpses; Mann–Whitney *U* test, P < 1.0E-15). Figure 2 shows the relationship between the mean probability of a drop and cluster size in the two models. In the Interaction Model, the probability of a drop gradually increased as a function of cluster size. This was perhaps because the carrying agents did not always drop a corpse with high probability even when the cluster size in front of them was large. In contrast, the probability in the Threshold-only Model suddenly increased at a cluster size of about 10, perhaps coincident with the threshold value. According to a previous report of ant experiments, the probability of dropping a corpse appeared to increase as cluster size increases^13^. The probability in the Interaction Model did not increase suddenly as in the Threshold-only Model and so better approximated the observed probability of dropping a corpse in the process of the formation of clusters of ant corpses.

**Figure 3:**
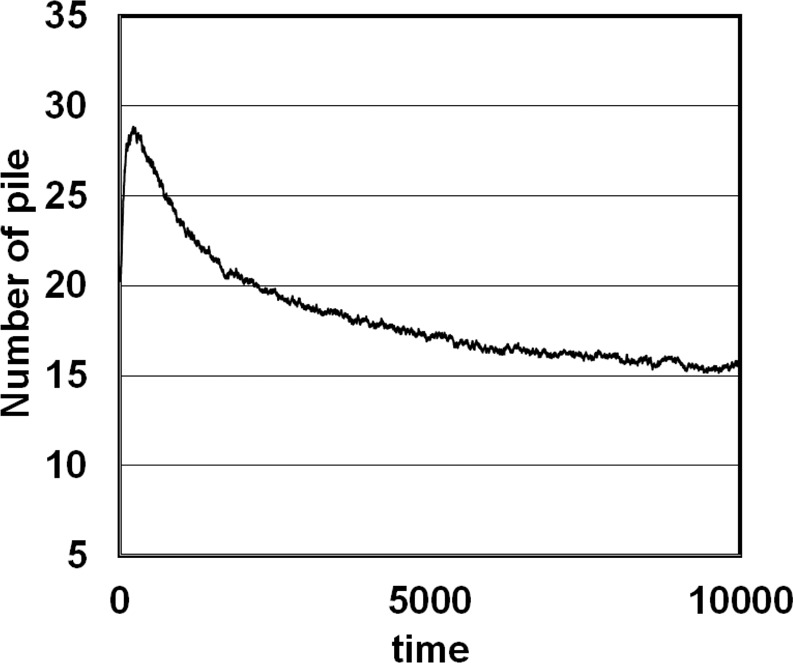
The time evolution of the mean number of piles (Interaction Model). The data are mean values from 100 trials.

**Figure 4:**
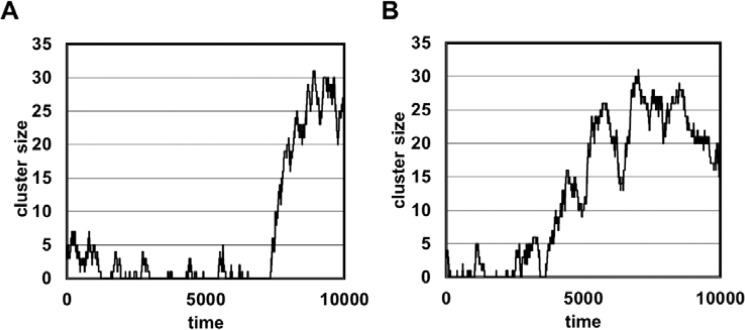
Examples of growth of a cluster of corpses (Interaction Model). Data from individual trials are shown. (**a**) The cluster grew in a very short time. (**b**) The cluster size continued to fluctuate to some extent.

Figure 3 shows the time evolution of the number of piles in the Interaction Model. Many piles are constructed in the initial stages, but these gradually disappear over time. This is consistent with the results of ant experiments, in which piles were observed to grow and others disappear, with the number falling after reaching a maximum value because the worker ants ‘pick up corpses from small piles and drop them to form a large pile’^13^. Similar results with ant experiments were also obtained in respect with the cluster formation. Figure 4 shows two examples of the growth of a cluster of corpses in the Interaction Model, which suggest that cluster formation is autocatalytic. Clusters of corpses grew rapidly over a short time span and reached a steady state, although cluster sizes sometimes repeatedly fluctuated to some extent before reaching the steady state (Fig. 4B).

**Figure 5:**
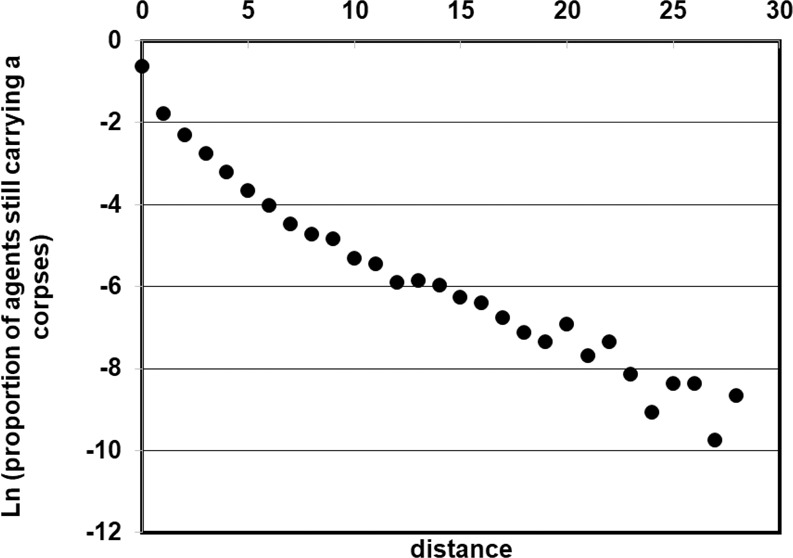
The proportion of agents that are still carrying a corpse at various distances (Interaction Model). The natural log of the proportion is plotted against distance, showing a near-linear relationship. Stacked data from 100 trials are shown.

Individual behaviours of carrying agents in the Interaction Model were examined. The distance covered by agents after picking up a corpse approximates a linear function of the natural log of the proportion of agents (slope = −0.26, *R*^2^ = 0.95; Fig. 5). The agents typically carry the corpses for shorter distances and less frequently cover longer distances, perhaps because the dropping probability in the Interaction Model varies. This finding is also in agreement with ant experiments^13^; however, the distances covered in the model were smaller than those observed, perhaps because the model allowed the agents to move forward or in the reverse direction with equal probability. Worker ants reverse direction with a smaller probability^13^.

**Figure 6:**
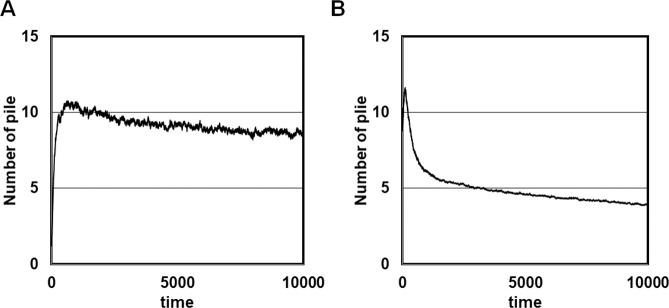
Effects of changes in the number of corpses (*φ*) and field length (*l*) on the time evolution of the mean number of piles (Interaction Model). In the main experiments, these were set as *φ* = 400 and *l* = 100. (**a**) Parameter values *φ* = 200 and *l* = 100. (**b**) Parameter values *φ* = 200 and *l* = 50.

Finally, the influence on the results of the number of corpses (*φ*) and the field length (*l*) was examined. Figure 6 shows the time evolution of the mean number of piles when the number of corpses was halved from 400 to 200 (Fig. 6A) and when both the field length was halved from 100 to 50 and the number of corpses was halved from 400 to 200 (Fig. 6B). Halving only the number of corpses (Fig. 6A) resulted in the disappearance of the sharp initial increase in the number of piles seen in Figs. 3 and 6B (which had the same ratios *φ*/*l*). Halving both the number of corpses and the field length (Fig. 6B) resulted in a rapid decrease in the number of piles after the initial maximum, but retained a similar evolution to that seen in Fig. 3. Interestingly, halving the density of corpses resulted in about half the number of piles present in the initial stage, i.e. up to maximum pile number (Fig. 3 vs. 6A). Furthermore, halving the field length while maintaining the same density of corpses resulted in approximately half the number of piles (Fig. 3 vs. 6B). These findings compare favourably with observations reported in previous research^13^.

## Discussion

This study investigated the growth of ant corpse piles by developing an agent-based model. In the model, the probability for whether an ant dropped the corpse it was carrying depended on whether there was another ant at the same location. The resulting model reproduced phenomena observed in the pile growth of real ants^13^. In the model, many small clusters of corpses were produced at an early stage, but after the number of piles reached a maximum value, some piles grew and others disappeared through a process of autocatalytic cluster formation.

Models in previous studies have generally assumed phenomenological results and functions directly obtained from experimental studies^2^. However, recent studies of macro-behaviours have used a simple threshold rule rather than a complicated function^29,30^. Previous models have hypothesised that ants can accurately detect the number of corpses within their detection areas. In our models, the agents did not need to accurately detect the number of corpses within their corpses, which is a different assumption from previous studies. However, as shown in results obtained from the Threshold-only Model, it is not enough for the agents to use only a simple threshold rule. Agent–agent interactions, namely nest–mate presence, will enable agents to exhibit a more complex pattern at the macro-level. On the other hand, in the Interaction Model, the carrying agents do not always drop the corpse just because the local cluster of corpses exceeds the threshold, which might result in facilitating the growth of specific clusters and inhibiting the growth of others. Therefore, agent–agent interactions will make a difference with respect to the probability of dropping a corpse between these two models. In fact, this probability in the Interaction Model appears to reproduce the observed probability of dropping a corpse in the process of the formation of clusters of ant corpses. Although the agents randomly walk, their spatial distribution can be unbalanced, triggering the effect of nest–mate presence on the probability of drop. Therefore, nest–mate presence might be an important piece of information for ant workers to determine whether the pile growth is stable or temporal. This is because they cannot recognise whether a corpse pile at their location will grow without pause or decrease as time goes on. Some corpses within a pile may be carried away by agents before the pile has grown in size, whereas those that are not fully grown can become fragmented. Ant workers may present complex and collective patterns by effectively using such information combined with stigmergy.

In that regard, the present model may describe how to model the decision-making processes of ants involved in the growth of ant corpse piles. Although there is no direct evidence that nest–mate presence has an influence on the coordination of the probability of dropping corpses, such an effect may bridge the gap between threshold response at the micro-level and the spatial macro-pattern of ant cemeteries. In fact, the growth of ant cemeteries and their pattern formation seem to be influenced by the surrounding environment^31,32^; because individual ants adjust their behaviours to the environment. Furthermore, the performance of worker ants can be increased or decreased according to interactions with their nest-mates^20^. In the present model, the contribution of each ant to the construction of clusters was influenced by whether other ants were present at the cluster location. Such a strategy combined with stigmergy may explain the construction and the formation of ant cemeteries. Nest–mate interactions are known to regulate and facilitate some building activities in insects^22,23^, and similar behaviours have been observed in other aggregation behaviours by ants, such as bridge formation^33,34^. The varying contribution of individual ants may therefore be a key effect that explains the dynamics of ant cemeteries and their activity.

## Supplementary Material

Supplementary Material.

## Data Availability

All data are available in the text and supplementary files.
