# Supplementary material for: Interactions between worker ants may influence the growth of ant cemeteries
Source: Sci Rep. 2020 Feb 11;10:2344. doi: 10.1038/s41598-020-59202-0 (PMC7012894; doi:10.1038/s41598-020-59202-0)
Supplement: Supplementary file 1 — Supplementary Material. [file 41598_2020_59202_MOESM1_ESM.docx]

**Interactions between worker ants may influence the growth of ant cemeteries**

**Tomoko Sakiyama**

**Supplementary materials**

**Pseudocode for the models**

*Interaction Model*

STEP 1: Corpse detection

STEP 2: Agent detection

STEP 3: Probability calculation for the Interaction Model

STEP 4: Picking up or dropping the corpse

STEP 5: Update the position

STEP 6: Update the time and go to STEP 1

*Threshold-only Model*

STEP 1: Corpse detection

STEP 2: Probability calculation for the Threshold-only Model

STEP 3: Picking up or dropping the corpse

STEP 4: Update the position

STEP 5: Update the time and go to STEP 1

*Corpse detection:*

The agent *k* can ‘detect’ a corpse only if that corpse is located at agent *k*’s current position *x^k^_t_*. At that time, the following parameters are set:

*corpse_sum^k^_t_* is reset to 0.

if a corpse is located at *x^k^_t_*,

then *corpse*_*detect^k^_t_* → 1 and calculates *corpse_sum^k^_t_*, (1)

else *corpse*_*detect^k^_t_* → 0. (2)

Here, *x^k^_t_* represents the current position of the agent *k* at time *t*, and *corpse*_*detect^k^_t_* determines whether or not the agent *k* detects a corpse at time *t*. The parameter *corpse_sum^k^_t_* represents the total number of corpses detected by the agent *k* at time *t*.

*Agent detection (Interaction Model only):*

Note: This step is not included in the Threshold-only Model.

The agent *k* can “detect” another agent only if that agent is located at the current position of agent *k*. The following parameters are set:

if an another agent is located at *x^k^_t_*, then *agent*_*detect^k^_t_* → 1, (3)

else *agent*_*detect^k^_t_* → 0. (4)

Here, *agent*_*detect^k^_t_* determines whether or not agent *k* detects another agent at time *t*.

*Probability calculation for the Interaction Model:*

Note: The probability calculation depends on the model. The calculation for the Threshold-only Model is given below.

The probability of agent *k* picking up or dropping a corpse is determined as follows:

if *carry^k^_t_* = 1 AND *corpse*_*detect^k^_t_* = 1,

if *agent_detect^k^_t_* = 1,

if *corpse_sum^k^_t_* != 0 AND *corpse_sum^k^_t_* < *threshold*,

then *prob^k^_t_* = *probability*_low_, (5)

else if *corpse_sum^k^_t_* >= *threshold,*

then *prob^k^_t_* = *probability*_high_. (6)

else if *agent_detect^k^_t_* = 0,

if *corpse_sum^k^_t_* != 0 AND *corpse_sum^k^_t_* < *threshold*,

then *prob^k^_t_* = *probability*_high_, (7)

else if *corpse_sum^k^_t_* >= *threshold,*

then *prob^k^_t_* = *probability*_low_. (8)

else if *carry^k^_t_* = 0 AND *corpse*_*detect^k^_t_* = 1,

then *prob^k^_t_* = *probability*_pick_ (9)

Here, *carry^k^_t_* specifies whether the agent *k* carries a corpse or not at time *t*, and *prob^k^_t_* indicates the probability of agent *k* dropping or picking up a corpse at time *t*.

If agent *k* is carrying a corpse (*carry^k^_t_* = 1) and there is at least one corpse at its current location, the probability of dropping the carried corpse (*probability*_high_ or *probability*_low_) depends on the relationship between *corpse_sum^k^_t_* and *threshold*, as well as on whether another agent has been detected (if not, *probability*_high_ or *probability*_low_ are reversed). Conversely, if agent *k* is not carrying a corpse (*carry^k^_t_* = 0) and detects a corpse at the current location, then the probability of picking up the corpse is (*probability*_pick_). If two or more agents are eligible to pick up a corpse, one of this is chosen randomly.

*Probability calculation for the Threshold-only Model:*

The probability of agent *k* picking up or dropping a corpse is determined as follows:

if *carry^k^_t_* = 1 AND *corpse*_*detect^k^_t_* = 1,

if *corpse_sum^k^_t_* != 0 AND *corpse_sum^k^_t_* < *threshold*,

then *prob^k^_t_* = *probability*_low_, (10)

else if *corpse_sum^k^_t_* >= *threshold,*

then *prob^k^_t_* = *probability*_high_. (11)

else if *carry^k^_t_* = 0 AND *corpse*_*detect^k^_t_* = 1,

then *prob^k^_t_* = *probability*_pick_ (12)

*Picking up or dropping the corpse:*

In the following, *rn^k^_t_* is a random number for which *rn^k^_t_* ∊ [0.0, 1.0].

if *corpse*_*detect^k^_t_* = 1,

if *carry^k^_t_* = 1 AND *rn^k^_t_* < *prob^k^_t_*,

agent *k* drops the corpse and *carry^k^_t_* → 0, (13)

else if *carry^k^_t_* = 0 AND *rn^k^_t_* < *prob^k^_t_*,

agent *k* picks up the corpse and *carry^k^_t_* → 1, (14)

else if *corpse*_*detect^k^_t_* = 0,

if *carry^k^_t_* = 1 AND *rn^k^_t_* < *prob_drop_*,

agent *k* drops the corpse and *carry^k^_t_* → 0 (15)

Equations (13) and (14) show that agent *k* drops or picks up a corpse based on the calculated probability *prob^k^_t_*. Equation (15) shows that agent *k* drops the corpse it is carrying with a constant (small) probability *prob_drop_* even if no corpses are detected at the current location.

*Update the position:*

Agent *k*’s current position is updated as follows:

*x^k^_t_* → *x^k^_t_* +1 or *x^k^_t_* – 1 with equal probability. (16)

This same action is performed fo all the agents.

*Update the time:*

*t* → *t*+1, for all agents. (17)
